# Development and Validation of Ferroptosis-Related lncRNAs as Prognosis and Diagnosis Biomarkers for Breast Cancer

**DOI:** 10.1155/2022/2390764

**Published:** 2022-10-18

**Authors:** Zhi-Yong Yao, Chaoqun Xing, Shanshan Cai, Xiao-Liang Xing

**Affiliations:** ^1^School of Public Health and Laboratory Medicine, School of Basic Medicine, The First Affiliated Hospital of Hunan University of Medicine, Hunan University of Medicine, Huaihua, 418000 Hunan, China; ^2^School of Nursing, Youjiang Medical College for Nationalities, Baise, 533000 Guangxi, China

## Abstract

Breast cancer (BC) is one of the most common malignancies affecting women. Ferroptosis is a novel cancer treatment option. The present study is aimed to identify suitable ferroptosis-related lncRNAs to predict and diagnose BC. Differential expression and Cox regression analyses were used to screen suitable prognostic biomarkers and construct a suitable risk model. We identified four ferroptosis-related differentially expressed lncRNAs (FR-DELs) (LINC01152, AC004585.1, MAPT-IT1, and AC026401.3), which were independently correlated with the overall survival of BC patients. The area under the curve value of the prognostic model using those four biomarkers was over 0.60 in all three groups. The sensitivity and specificity of the diagnostic model using those four biomarkers were 86.89% and 86.73%, respectively. Our present study indicated that these four FR-DELs (LINC01152, AC004585.1, MAPT-IT1, and AC026401.3) could be prognostic biomarkers for BC, although clinical validation studies are required.

## 1. Introduction

With more than 2 million new cases (11.7% of all new cancer cases) and 68 thousand deaths (6.9% of all cancer deaths) in 2020 globally, breast cancer (BC) is one of the most common malignancies in women [1]. With the development of early screening and anticancer strategies, the treatment effect of BC has improved dramatically [2]. However, the recurrence rates of BC remain high [3, 4]. Additionally, evidence has indicated that BC's prognosis is affected by many factors, such as age, tumor size, grade, lymph node involvement, and lymphatic vascular infiltration [5, 6]. The prognosis of BC remains a complex problem, even though many BC prognostic biomarkers have been discovered in ESMO Clinical Practice Guidelines for diagnosis, treatment, and follow-up [5–13]. Constructing a new risk assessment model to predict the prognosis of BC patients is necessary.

The main treatments for BC are surgery with adjuvant radiation therapy and chemotherapy [14–18]. However, many BC patients are resistant to chemotherapy, endocrine, and targeted therapy, and these patients generally have poor survival rates [6, 19–24]. The complexity and high heterogeneity of the underlying mechanisms of BC make its treatment difficult [25–29]. Ferroptosis is a novel type of cell death that plays an essential role in the progression of many diseases, including cancers [30]. Intriguingly, previous studies have indicated that erastin, a ferroptosis inducer, could enhance the effectiveness of chemotherapy drugs [31–34]. These results suggest that ferroptosis may be a novel anticancer therapeutic strategy.

Long noncoding RNAs (lncRNA) are a class of transcripts with 200 nucleotides in length, which generally do not have protein-coding potential. Previous evidence demonstrated that lncRNAs play an essential role in various biological processes, including the proliferation, apoptosis, migration, and invasion of cancer cells [35–38]. Many lncRNAs are closely related to the survival status and could be prognostic biomarkers for several cancers [39–41]. Therefore, we aimed to identify suitable ferroptosis-related lncRNAs as prognostic biomarkers to predict the outcome of BC.

## 2. Materials and Methods

### 2.1. Data Acquisition

The RNAseq data (counts) and their corresponding clinical information for 1215 samples (113 normal and 1102 BC patients) were downloaded from the Cancer Genome Atlas database (https://portal.gdc.cancer.gov/projects/TCGA-BRCA). The annotation lncRNAs and recognized ferroptosis-related genes were downloaded from the Gene Transfer Format file [42]and FerrDb [43], respectively. Using the Estimate script in R (3.6.1), we used all normalized gene expression values to evaluate Stromal Score, Immune Score, Tumor Purity, and Estimate Score. The evaluated infiltrating score of immune cells and immune factors were downloaded from Tumor IMmune Estimation Resource (TIMER).

### 2.2. Differentially Expressed Analyses

DESeq2 in R (3.6.1) was used to screen the differentially expressed genes with the specific criterion as follows: adj. *p* < 0.05, |logFC| ≥ 0.5, and basemean ≥ 50. The ferroptosis-related DEGs (FR-DEGs) were obtained by overlapping with the recognized ferroptosis related-genes. The ferroptosis-related lncRNAs (FR-DELs) were obtained by correlation analyses of FR-DEGs with DELs with the following criteria: *p* < 0.05 and *r* > 0.5.

### 2.3. Survival Analyses and Principal Component Analyses

After regrouping the samples by the median value of each gene, we performed univariate and multivariate Cox regression analyses using survival, survminer, and regparallel packages in R software (3.6.1). We carry out the principal component analyses (PCA) in R software (3.6.1).

### 2.4. Construction of Prognostic and Diagnosis Model

1. Prognostic model, Risk value = Express_LINC01152_^∗^ *β*_LINC01152_ + Express_AC004585.1_^∗^ *β*_AC004585.1_ + Express_MAPT−IT1_^∗^ *β*_MAPT−IT1_ + Express_AC026401.3_^∗^ *β*_AC026401.3_. The expression values were obtained from the normalized value of DESeq2 analyses, and the *β* were obtained from multivariate Cox regression analyses [44]
2. Diagnosis model, The Logit value = Constant + Express_LINC01152_^∗^ *β*_LINC01152_ + Express_AC004585.1_^∗^ *β*_AC004585.1_ + Express_MAPT−IT1_^∗^ *β*_MAPT−IT1_ + Express_AC026401.3_^∗^ *β*_AC026401.3_. The Express (Exp) values were obtained from the normalized value of DESeq2 analyses, and the constant and *β* were obtained from a stepwise logistic regression analysis [44]

### 2.5. Statistical Analyses

Unpaired two-tailed Student's *t*-test was used to investigate the relationship of risk value with the clinical characteristics. Time-dependent receiver operating characteristic (ROC) curves were used to estimate the utility of the outcome prediction.

## 3. Results

### 3.1. Differential Expression Analyses

**Figure 1:**
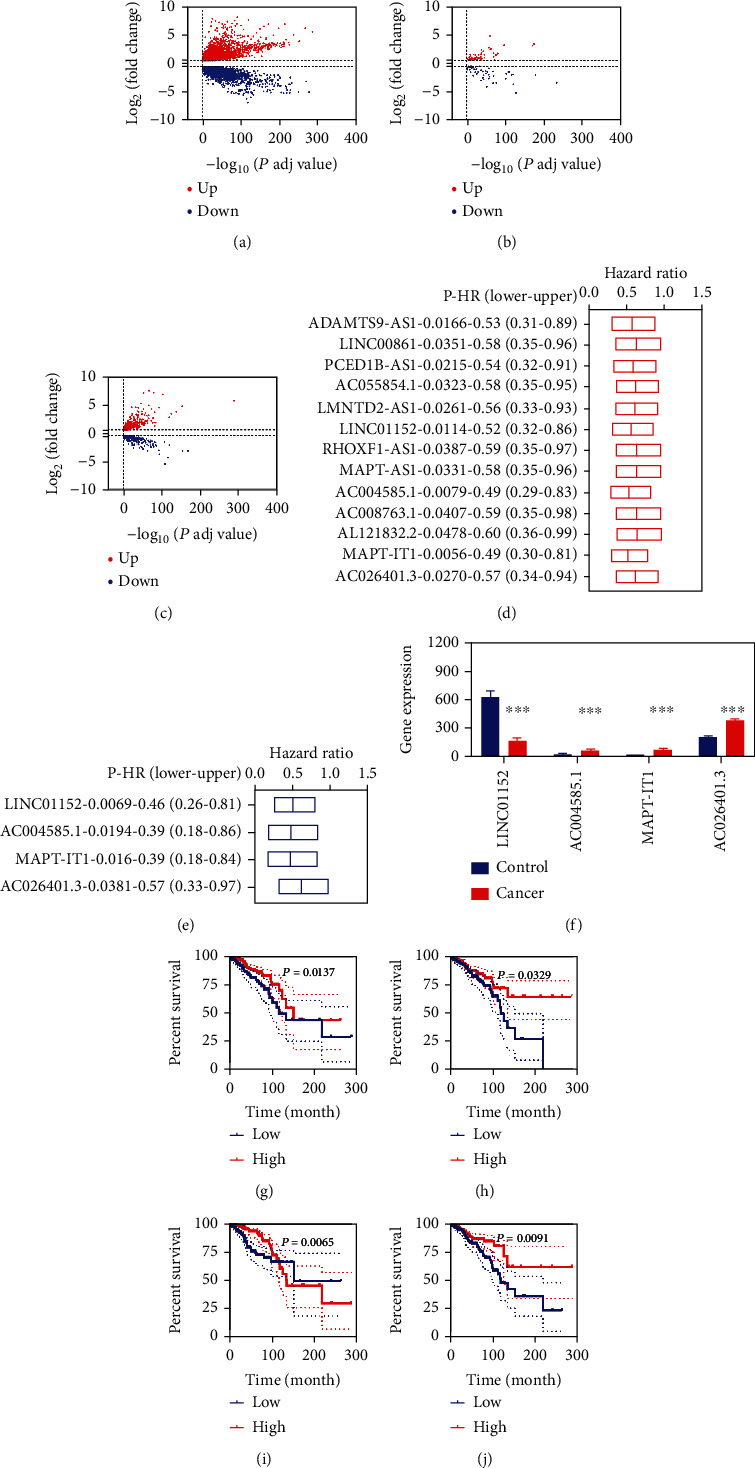
Differential expression analyses for BC. (a-c), differentially expressed genes (a), ferroptosis-related differentially expressed genes (b), and differentially expressed lncRNAs (c) for BC. (d), univariate Cox regression illustrated 13 FR-DELs correlated with prognosis. (e), multivariate Cox regression illustrated four FR-DELs correlated with prognosis. (f), expression of these four FR-DELs between control and BC. (g-j), survival curve of these four FR-DELs in the training group. (g), LINC01152. (h), AC004585.1.(i), MAPT-IT1. (j), AC026401.3.

Through differentially expressed analyses, we obtained 8495 DEGs, including 5337 upregulated DEGs and 3158 downregulated DEGs (Figure 1(a)). By overlapping analyses of DEGs with ferroptosis genes, we obtained 119 FR-DEGs (65 upregulated DEGs and 54 downregulated DEGs) (Figure 1(b)). Through similar analyses, we obtained 709 DELs, including 430 upregulated DELs and 279 downregulated DELs (Figure 1(c)). To obtain FR-DELs, we introduced the Spearman correlation analyses for these 119 FR-DEGs and 709 DELs and obtained 787 pairs of FR-DEGs-DELs, including 74 FR-DEGs and 203 DELs. We named these 203 DELs as FR-DELs.

### 3.2. Development and Validation of Prognosis Biomarkers

To screen for suitable prognosis biomarkers and verify them, we randomly divided the BC patients into training and validation groups (Table 1). We found that 13 FR-DLEs (Figure 1(d)) and four FR-DELs (Figure 1(e)) were correlated with the overall survival (OS) of patients with BC by univariate and multivariate Cox regression analyses, respectively, in the training group. The expression of LINC01152 was significantly decreased, while AC004585.1, MAPT-IT1, and AC026401.3 increased significantly (Figure 1(f)). The Kaplan-Meier curves of those four FR-DELs were displayed in Figures 1(g)–1(j).

**Figure 2:**
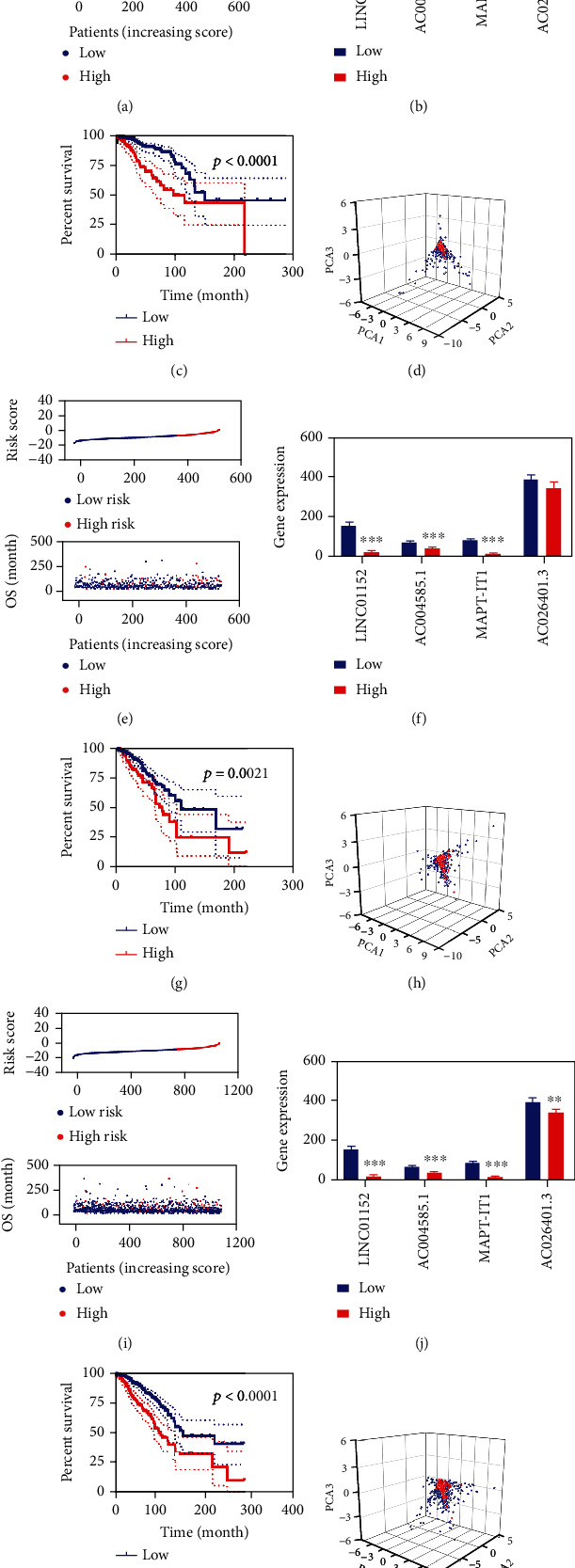
Development and validation of prognosis signature for BC. (a-d), risk value and survival status (a), expression (b), survival curve (c), PCA analyses (d) using these four FR-DELs in the training group. (e-h), risk value and survival status (e), expression (f), survival curve (g), PCA analyses (h) using these four FR-DELs in the validation group. (i-l), risk value and survival status (i), expression (j), survival curve (k), PCA analyses (l) using these four FR-DELs in the entire group. ^∗^*p* < 0.05, ^∗∗^*p* < 0.01, ^∗∗∗^*p* < 0.001.

We used these four FR-DELs to construct a risk assessment model in the training, validation, and entire group. The Youden index (cutoff value = −15.25) in the training group was used to regroup the patients with BC into low and high-risk groups (Supplementary Figure 1). The risk value and survival status for each patient in the training group were displayed in Figure 2(a). The expressions of these four FR-DELs were significantly decreased in patients with BC with high-risk values (Figure 2(b)). The BC patients with high-risk values displayed worse OS (Figure 2(c)).

Subsequently, we performed similar analyses in the validation and entire groups. These results were shown in Figures 2(e)–2(l). PCA analyses indicated that these BC patients with high-risk values could well be distinguished from the BC patients with low-risk values using these four FR-DELs (Figures 2(d), 2(h), and 2(l)).

### 3.3. Independent Prognostic Factors of Overall Survival

**Figure 3:**
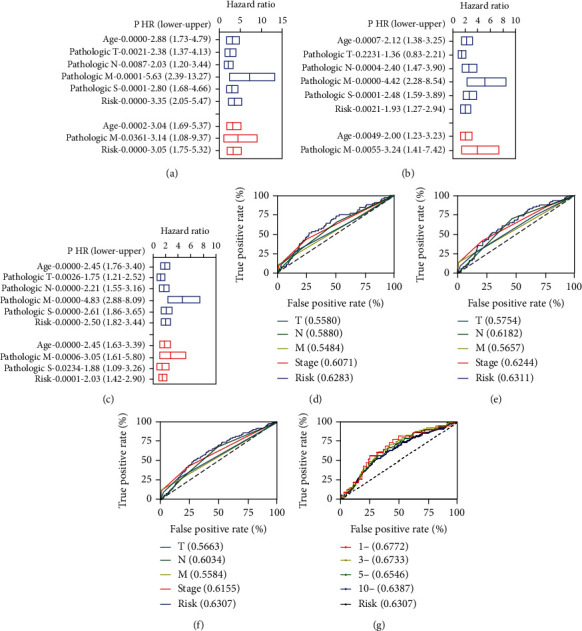
Prognostic factors of clinical features for BC. (a-c), univariate (blue) and multivariate (red) Cox regression analyses for clinical features and the risk model in the training (a), validation (b), and entire group (c), respectively. (d-f), comparison of ROC curve of risk model with the clinical features. (d), for patients in the training group. (e), for patients in the validation group. (f), for patients in the entire group. (g), time-dependent ROC curve of the risk model in the entire group.

To know whether the risk model could be used independently, we performed univariate and multivariate Cox regression analyses for different clinical features and the risk model. We found that age, pathological TNM, pathological Stage, and risk model were correlated with the OS, whereas age, pathological M, and risk model were correlated with the OS independently (Figure 3(a)). In the validation group, the age, pathological NM, pathological Stage, and risk model were correlated with the OS by univariate Cox regression analyses. In contrast, age and pathological M were correlated with the OS by multivariate Cox regression analyses (Figure 3(b)). In the entire group, the age, pathological NM, pathological Stage, and risk model were correlated with the OS by univariate Cox regression analyses, whereas the age, pathological M, pathological Stage, and risk model were correlated with the OS by multivariate Cox regression analyses (Figure 3(c)). Compared with pathological TNM and Stage, these results suggested that these four FR-DELs were more accurate as measured by the ROC analyses (Figure 3(d)–3(f)). The area under curve (AUC) value of the prognostic model using those four biomarkers was over 0.60 in all three groups. Subsequently, we performed ROC curves analyses at 1, 3, 5, and 10-year in the entire group (Figure 3(g)).

**Figure 4:**
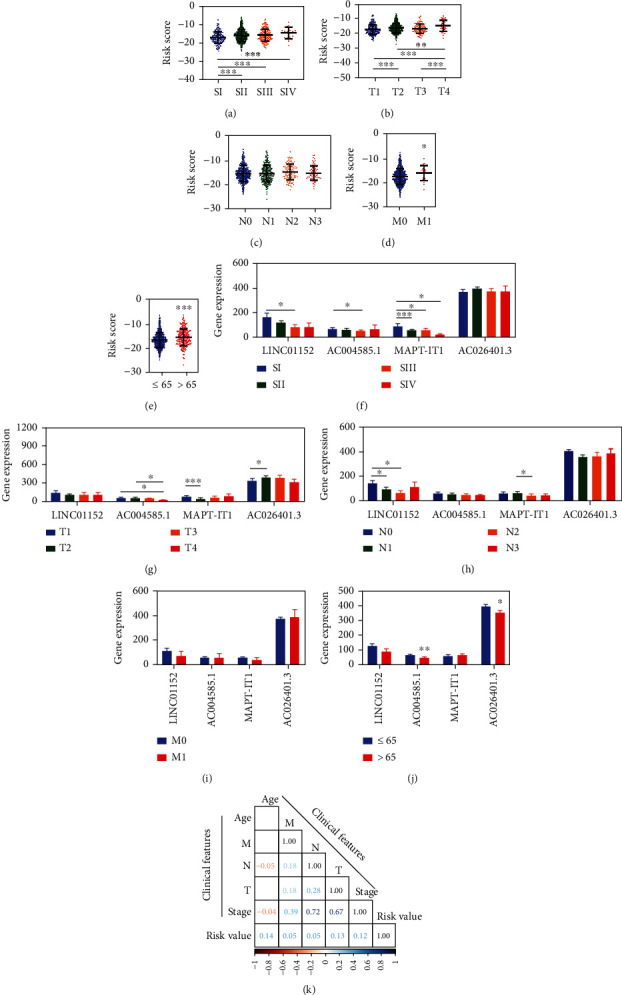
Correlation of risk value and prognosis biomarkers with clinical features. (a-e), correlation of risk value with clinical features. (a), for the pathologic Stage. (b), for pathologic T. (c), for pathologic N. (d), for pathologic M. (e), for age. (f-j), correlation of expression of these four FR-DELs with clinical features. (f), for the pathologic Stage. (g), for pathologic T. (h), for pathologic N. (i), for pathologic M. (j), for age. (k), correlation analyses for the risk model with the clinical features. ^∗^*p* < 0.05, ^∗∗^*p* < 0.01, ^∗∗∗^*p* < 0.001.

To know the relationship of the risk model with clinical features, we performed comparative analyses for risk value in different groups for the entire group. The results were displayed in Figures 4(a)–4(e). Then, we performed comparative analyses for these four FR-DELs in sets for the entire group. The results were shown in Figures 4(f)–4(j). Additionally, we performed the correlation analyses for the risk model with the different clinical features and found no significant correlation (Figure 4(k)).

### 3.4. Correlation Analyses with the Immunity

**Figure 5:**
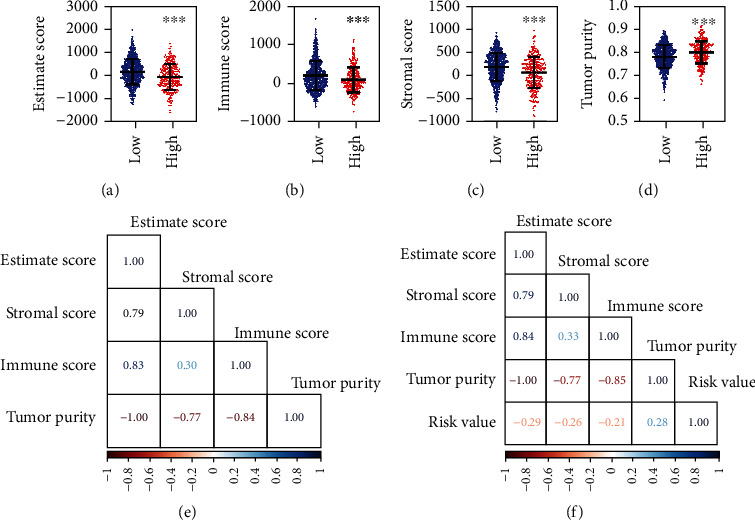
Comparison and correlation analyses of risk assessment model with the immunity. (a-d), comparison of the ESTIMATE score (a), immune score (b), stromal score (c), and tumor purity (d) between the patients with BC with high- and low-risk values. (e-f), correlation analyses for ESTIMATE score, immune score, stromal score, and tumor purity between the normal and patients with BC patients (e), between patients with BC with high- and low-risk value (f). ^∗∗∗^*p* < 0.001.

Previous studies demonstrated that ferroptosis and the immune system could regulate each other to achieve their antitumor effect. We used the ESTIMATE package to evaluate the immune status. We found that the ESTIMATE score and stromal score were significantly decreased while the immune score and tumor purity were significantly increased in the patients with BC (Supplementary Figures 2(a)–2(d)). In the patients with BC with a high-risk value, the ESTIMATE score, immune scores, and stromal scores were significantly decreased while the tumor purity was significantly increased (Figure 5(a)–5(d)). The correlations of ESTIMATE score, immune score, stromal score, tumor purity, and risk value were displayed in Figures 5(e)–5(f).

**Figure 6:**
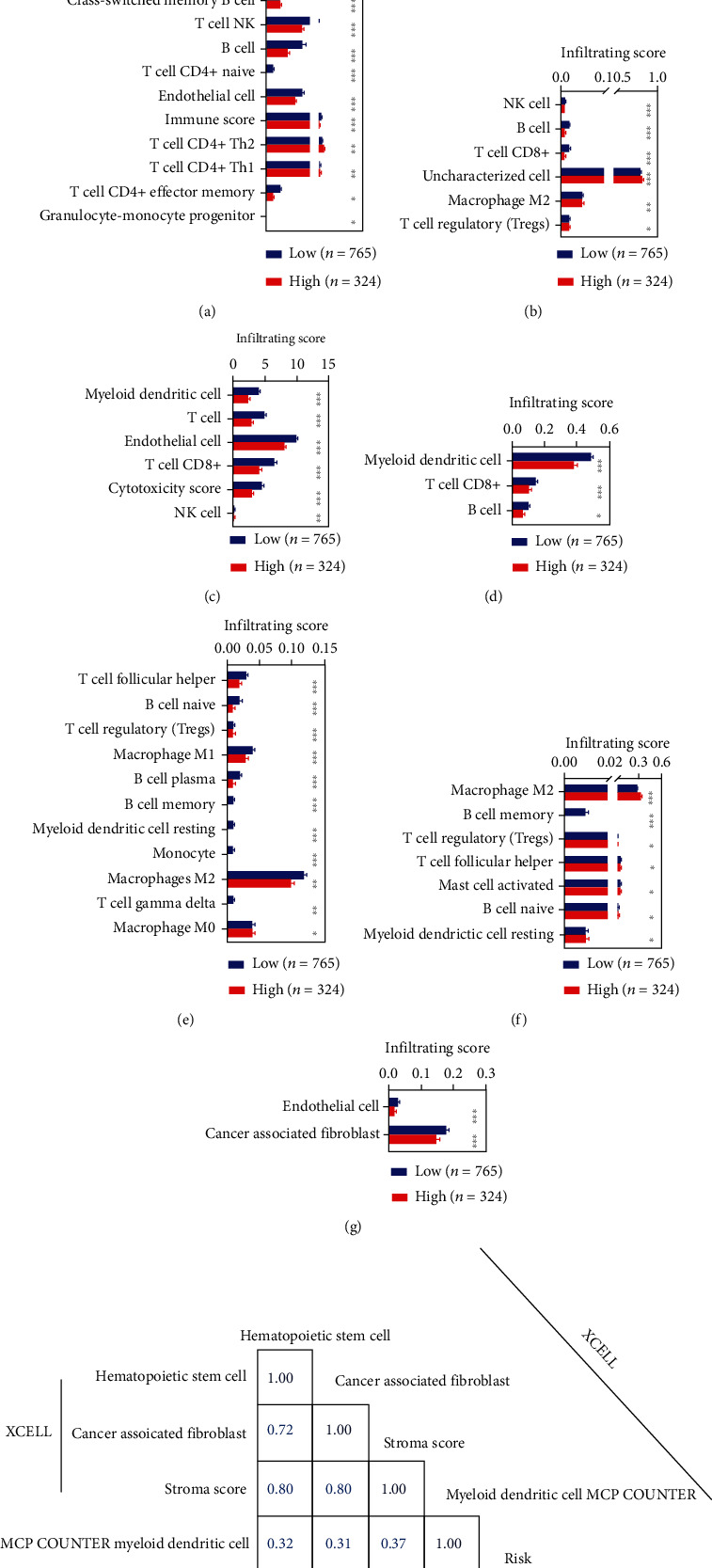
Correlation analyses of risk value with the immune infiltration. (a-g), differentially expression analyses of infiltrating score between BC patients with low-risk value and BC patients with high-risk value. (a), XCELL. (b), QUANTISEQ. (c), MCPCOUNTER. (d), TIMER. (e), CIBERSORT-ABS. (f), CIBERSORT. (g), EPIC. (h), the immune cells and factors significantly correlated with the risk model. ^∗^*p* < 0.05, ^∗∗^*p* < 0.01, ^∗∗∗^*p* < 0.001.

We also carried out the difference analyses for the infiltration of immune cells and immune factors of each BC patient. We found that 84 immune cells and factors differ between normal and BC patients. Of the 84, we found that 55 immune cells and factors differ significantly (Figures 6(a)–6(g)). We introduced the correlation analyses for the risk value and these 55 immune cells and factors. We found that four immune cells and factors were significantly correlated with the risk model in the entire group.

### 3.5. Construction of a Diagnostic Model

X-ray mammography is the golden standard for diagnosing BC patients, which has some drawbacks, such as unsuitability for people under 40, for people with high gland density, can be done no more than twice a year, and high cost [45]. Therefore, to know its role in diagnosing patients with BC, we performed a stepwise logistic regression analysis for these four FR-DELs and constructed a diagnosis model according to previous studies. The sensitivity and specificity of the diagnosis model were 86.89% and 86.73%, respectively (Table 2). We also plotted the ROC curve of the diagnosis model, and the AUC value was 0.9277 (Supplementary Figure 3).

## 4. Discussions

With the development of early screening and anticancer strategies, the treatment of BC has improved dramatically. However, the recurrence rates of BC remain high. Therefore, it is necessary to construct a new risk prediction model to predict the outcome. Ferroptosis is a novel type of cell death, which could be a novel anticancer therapeutic strategy. In the present study, we identified four FR-DELs (LINC01152, AC004585.1, MAPT-IT1, and AC026401.3) correlated with the OS of BC.

The ROC curve showed that the AUC value of the risk model constructed by these four FR-DELs was over 0.6, which indicated that these four FR-DELs (LINC01152, AC004585.1, MAPT-IT1, and AC026401.3) could be prognosis biomarkers for BC in the outcome prediction. Chen et al. found that LINC01152 is up-expressed in HBV-positive hepatocellular carcinoma (HCC) [46]. LINC01152 could promote HCC cell proliferation and tumor formation in nude mice [46]. Wu et al. found that LINC01152 was upregulated in glioblastoma multiforme [47]. LINC01152 could promote the progression of GBM by upregulating MAML2 [47]. But in the present study, we found that LINC01152 was downregulated, which differed from previous studies. The patients with BC with low expression of LINC01152 displayed worse OS. In the present study, we found that the expression of AC004585.1 was significantly increased in BC and significantly decreased in BC with a high-risk value. By the univariate and multivariate Cox regression analyses, we found that AC004585.1 was correlated with the OS of BC. Our present study further increased the possibility of AC004585.1 as a prognosis biomarker for BC [48, 49]. The expression of MAPT-IT1 was significantly increased in BC and significantly decreased in BC patients with a high-risk value. In the present study, we found that MAPT-IT1 was correlated with OS of BC significantly. The HR ratio of less than 1.0 was consistent with the previous study [50]. There have been few studies on the function of MAPT-IT1, but our results further confirmed the possibility of MAPT-IT1 as a prognosis biomarker for BC. The expression of AC026401.3 was significantly increased in BC. We found AC026401.3 was correlated with the OS of BC. The HR ratio was less than 1.0. The patients with high expression of AC026401.3 displayed better OS. Our present study indicated that AC026401.3 could be a prognosis biomarker for BC. Previous studies also demonstrated that AC026401.3 was correlated with the OS and could be a prognosis biomarker for several cancers, including renal adenocarcinoma, colon adenocarcinoma, and ovarian cancer. These results indicated that AC026401.3 might play an essential role in developing a variety of cancers.

Although imaging diagnoses have high sensitivity and specificity, these methods also have some major drawbacks, such as being unsuitable for people under 40, for people with high gland density, and can be done no more than twice a year [45]. Although the sensitivity and specificity of this model were lower than those of imaging diagnosis, molecular biotechnology examination can overcome these shortcomings. LncRNA can regulate the cell cycle of tumor cells and various cell signaling pathways of cancer cell proliferation, apoptosis, migration, and invasion of cancer cells [35–38], which could be prognostic and diagnostic biomarkers for several cancers [39–41]. Such as Ye et al. found that the AUC value of myocardial infarction associated transcript as a diagnostic biomarker was 0.86, and the sensitivity and specificity were 87.80% and 75.61%, respectively [51]. El-Ashmawy et al. found that lncRNA-ATB had a high AUC value (AUC: 0.844, *p* < 0.01) for early diagnosis of breast cancer in patients with stage I-II [52]. In our present study, we found that the AUC value of the diagnostic model reached 0.927. The sensitivity and specificity of this diagnostic model were 86.89% and 86.73%, respectively. These results suggest that this model may be a better diagnostic model for breast cancer, although further studies are needed to verify it. Additionally, although the sensitivity and specificity of this model were lower than those of imaging diagnosis, it can overcome these shortcomings.

## 5. Conclusions

In the present study, we found four FR-DELs (LINC01152, AC004585.1, MAPT-IT1, and AC026401.3) that could be used as prognosis and diagnosis biomarkers to predict the outcome of BC. Our study provides a new perspective on the prognosis and diagnosis of BC, although there were several limitations, especially due to the lack of clinical validation.

## Figures and Tables

**Table 1:** Clinical features of BC in different groups.

Clinical features	Entire (*n* = 1091)	Training (*n* = 546)	Validation (*n* = 545)
Age			
≤65	772	388	384
>65	319	158	161
Gender			
Female	1079	539	540
Male	12	7	5
Stage			
Stage I	181	87	94
Stage II	619	312	307
Stage III	248	133	115
Stage IV	20	7	13
Unknown	23	7	16
T			
T1	279	133	146
T2	631	321	310
T3	138	71	67
T4	40	20	20
TX	3	1	2
N			
N0	514	252	262
N1	361	187	174
N2	120	60	60
N3	76	40	36
Unknown	20	7	13
M			
M0	908	456	452
M1	22	8	14
Unknown	161	82	79
Vital			
Alive	939	482	457
Death	152	64	88

**Table 2:** Sensitivity and specificity of diagnosis model.

	Real cancer	Real normal
Predicted cancer	948	15
Predicted normal	143	98
Total	1091	113
Correct	948	98
Sensitivity	0.8689	
Specificity		0.8673

## Data Availability

The data that support the findings of this study are openly available in TCGA at https://portal.gdc.cancer.gov/.

## Supplementary Materials

Supplementary Materials Supplementary Figure 1: youden index from the training group. Supplementary Figure 2: comparison analyses of risk assessment model of the ESTIMATE score between normal and cancer. Supplementary Figure 3: ROC curve of the diagnosis model.
