# Supplementary material for: Development and Validation of Ferroptosis-Related lncRNAs as Prognosis and Diagnosis Biomarkers for Breast Cancer
Source: Biomed Res Int. 2022 Oct 18;2022:2390764. doi: 10.1155/2022/2390764 (PMC9596248; doi:10.1155/2022/2390764)
Supplement: Supplementary Materials — Supplementary Figure 1: youden index from the training group. Supplementary Figure 2: comparison analyses of risk assessment model of the ESTIMATE score between normal and cancer. Supplementary Figure 3: ROC curve of the diagnosis model. [file 2390764.f1.zip › Supplementary_2022.10.01.docx]

**Development and Validation of Ferroptosis-related LncRNAs as Prognosis and Diagnosis Biomarkers for Breast Cancer**

**Zhi-Yong Yao^1#^, Chaoqun Xing^1,2#^, Shanshan Cai^1^, Xiao-Liang Xing^1*^**

^1^School of Public Health and Laboratory Medicine, School of Basic Medicine, The First Affiliated Hospital of Hunan university of Medicine, Hunan University of Medicine, Huaihua 418000, Hunan P. R. China.

^2^School of Nursing, Youjiang Medical College for Nationalities, Baise 533000, Guangxi P. R. China.

^#^Contributed equally to this work.

^*^Corresponding author: Xiao-Liang Xing, xiaoliangxinghnm@126.com.

**Supplementary information:** 3 figures


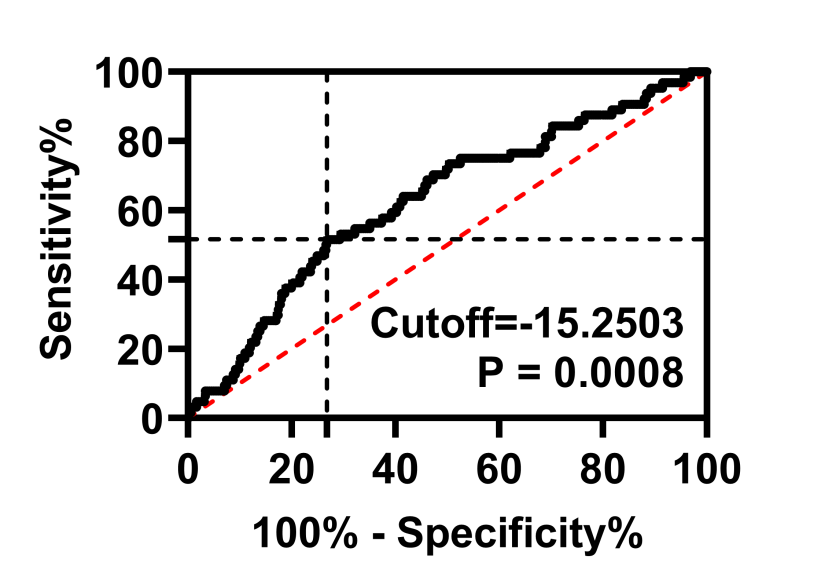


**Supplementary Figure 1 Youden index from the training group**


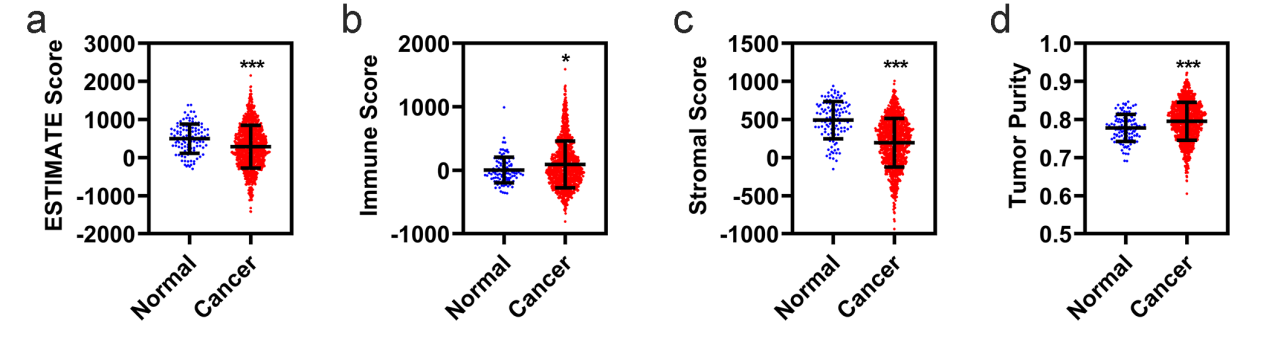


**Supplementary Figure 2 Comparison analyses of risk assessment model of the ESTIMATE score between normal and cancer.**


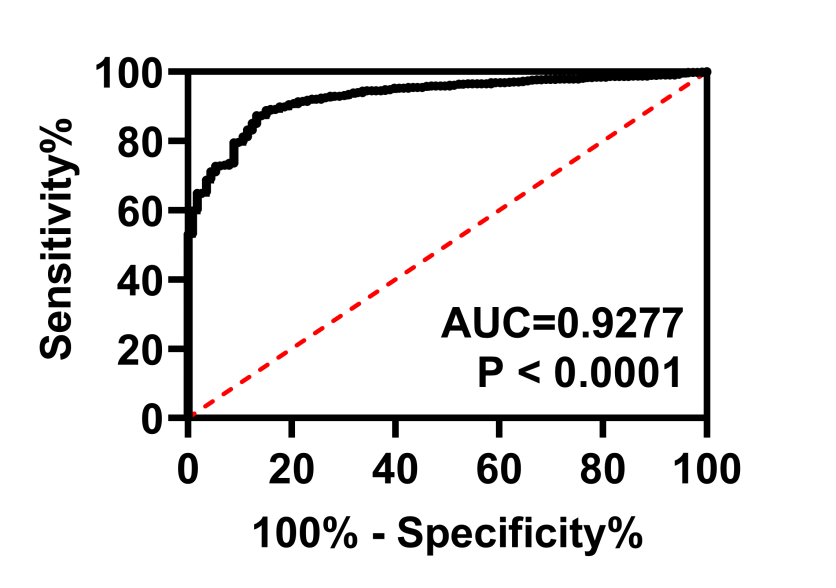


**Supplementary Figure 3 ROC curve of the diagnosis model.**
