# Supplementary figures and images for: Development and Validation of Ferroptosis-Related lncRNAs as Prognosis and Diagnosis Biomarkers for Breast Cancer
Source: Biomed Res Int. 2022 Oct 18;2022:2390764. doi: 10.1155/2022/2390764 (PMC9596248; doi:10.1155/2022/2390764)

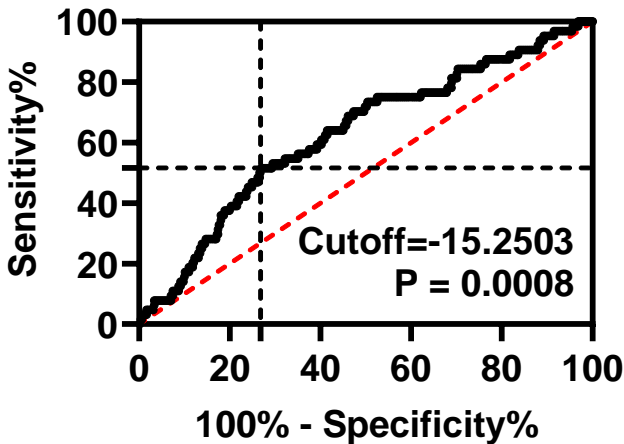

Supplement: Supplementary Materials — Supplementary Figure 1: youden index from the training group. Supplementary Figure 2: comparison analyses of risk assessment model of the ESTIMATE score between normal and cancer. Supplementary Figure 3: ROC curve of the diagnosis model. [file 2390764.f1.zip › Supplementary figure 1 (1).pdf]

**a**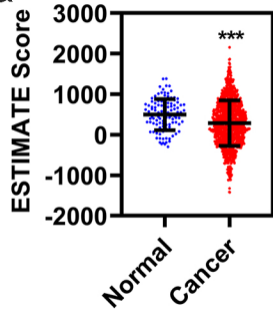**b**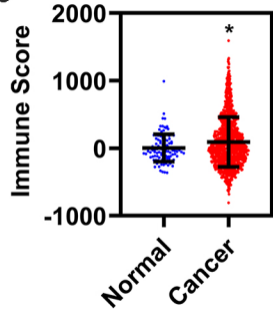**c**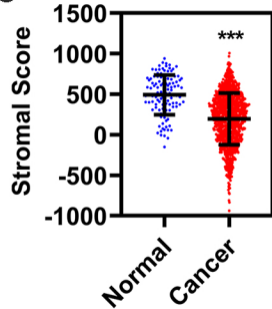**d**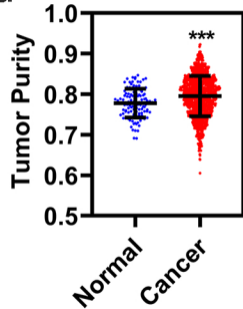

Supplement: Supplementary Materials — Supplementary Figure 1: youden index from the training group. Supplementary Figure 2: comparison analyses of risk assessment model of the ESTIMATE score between normal and cancer. Supplementary Figure 3: ROC curve of the diagnosis model. [file 2390764.f1.zip › Supplementary figure 2 (1).pdf]

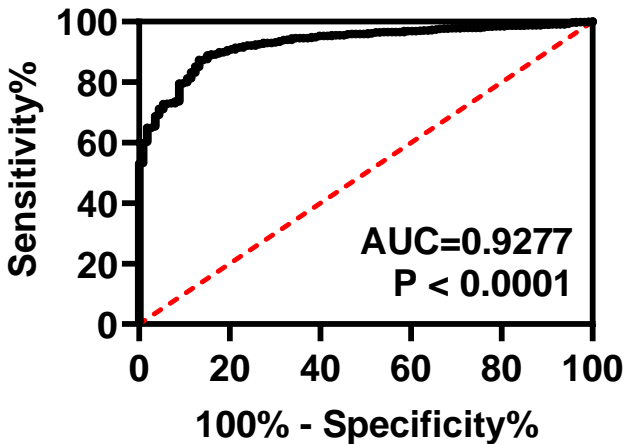

Supplement: Supplementary Materials — Supplementary Figure 1: youden index from the training group. Supplementary Figure 2: comparison analyses of risk assessment model of the ESTIMATE score between normal and cancer. Supplementary Figure 3: ROC curve of the diagnosis model. [file 2390764.f1.zip › Supplementary figure 3 (1).pdf]
